# The Prolyl Isomerase Pin1 Controls Lipopolysaccharide-Induced Priming of NADPH Oxidase in Human Neutrophils

**DOI:** 10.3389/fimmu.2019.02567

**Published:** 2019-11-01

**Authors:** Min Liu, Samia Bedouhene, Margarita Hurtado-Nedelec, Coralie Pintard, Pham My-chan Dang, Shiyuan Yu, Jamel El-Benna

**Affiliations:** ^1^College of Chemical Engineering, Nanjing Forestry University, Nanjing, China; ^2^INSERM U1149, ERL 8252 CNRS, Centre de Recherche sur l'Inflammation, Université Paris Diderot, Sorbonne Paris Cité, Laboratoire d'Excellence Inflamex, Faculté de Médecine, Site Xavier Bichat, Paris, France; ^3^Yitong Food Industry Co., Ltd, Xuzhou, China; ^4^Laboratoire de Biochimie Appliquée et de Biotechnologie, Faculté des Sciences Biologiques et des Sciences Agronomiques, Université M. Mammeri, Tizi-Ouzou, Algeria; ^5^Departement d'Immunologie et d'Hématologie, Unité Dysfonctionnements Immunitaires, Centre Hospitalo-Universitaire Xavier Bichat, Paris, France

**Keywords:** neutrophils, LPS, Pin1, NADPH oxidase, NOX2, priming, ROS, p47^*phox*^

## Abstract

Production of superoxide anion and other reactive oxygen species (ROS) by neutrophils has a vital role in host defense against microbes. However, over-production can induce cell injury participating to inflammation. Superoxide anion is produced by the phagocyte NADPH oxidase/NOX2, a multicomponent enzyme system consisting of six proteins: two trans-membrane proteins (gp91^*phox*^ and p22^*phox*^) and four soluble cytosolic proteins (p40^*phox*^, p67^*phox*^, p47^*phox*^, and the small G-proteins, Rac1/2). Phosphorylation of p47^*phox*^ on several serines regulates NADPH oxidase activation. LPS released by gram negative bacteria can enhance or prime neutrophil superoxide production in combination with other agonists such as the bacterial peptide formyl-Met-Leu-Phe (fMLP). Since the pathways involved in LPS-induced priming are not completely understood, we investigated the role of the prolyl *cis/trans* isomerase Pin1 in this process. Two different Pin1 inhibitors, PiB, and Juglone are able to block LPS-induced priming of ROS production by human neutrophils in a concentration dependent manner. PiB and Juglone did not inhibit LPS-induced CD11b translocation neither CD62L shedding. LPS induced an increase of Pin1 activity in neutrophils similar to TNFα and fMLP. Since the phosphorylation of p47^*phox*^ on Ser345 is critical for NADPH oxidase up-regulation, we investigated the effect of LPS on this process. Results show that LPS induced the phosphorylation of p47^*phox*^ mainly on serine 345 and induced the activation of p38MAPKinase and ERK1/2. These results suggest that the prolyl *cis/trans* isomerase Pin1 may control LPS-induced priming of superoxide production in human neutrophils. Pharmacological targeting of Pin1 could be a valuable approach in sepsis.

## Introduction

Polymorphonuclear neutrophils (PMN) are the most abundant immune cells in human blood (1). PMN have a central role in host defense against pathogens and in inflammation (1, 2). Upon inflammation and infection, PMN are the first circulating cells to reach the inflammatory and infection site (3, 4). They are attracted by a variety of peptides, chemokines and lipids such as the C5a, N-formyl-methionyl-leucyl-phenylalanine (fMLF or fMLP), interleukin 8 (IL-8), LTB4, and platelet activating factor (PAF). Then they recognize microbes by their TLR receptors, engulf them and release huge number of anti-bacterial agents such as reactive oxygen species (ROS), myeloperoxidase, proteases, glucosidases, and anti-bacterial peptides in order to kill and eliminate microbes (3–6).

The enzyme responsible for ROS production is the nicotinamide adenine dinucleotide phosphate reduced form (NADPH) oxidase, also referred as NOX2 (7, 8); which produces superoxide anion (${\text{O}}_{2}^{•-}$), the source of other ROS molecules such as hydrogen peroxide (H_2_O_2_) which is used by myeloperoxidase to produce hypochlorous acid (HOCl), all of which cause the destruction and death of pathogens in the phagosome (6–9).

The phagocyte NADPH oxidase/NOX2 is a multicomponent enzyme system consisting of six proteins: two transmembrane proteins (gp91^*phox*^ and p22^*phox*^) and four soluble cytosolic proteins (p40^*phox*^, p67^*phox*^, p47^*phox*^, and the small G-proteins, Rac1/2) (7, 8). In resting cells, NOX2 is in a dormant state with spatial separation of the components. After neutrophil stimulation by different agents such as fMLP or phorbol myristate acetate (PMA), the cytosolic subunits p47^*phox*^, p67^*phox*^, and p40^*phox*^ are phosphorylated and migrate to associate with gp91^*phox*^ and p22^*phox*^ in the membrane to assemble the active NADPH oxidase (10). Phosphorylation of p47^*phox*^ on several serines (Ser303-379) located in the C-terminal portion of the protein plays an important role in NADPH oxidase activation (10, 11).

In addition to resting and activated state, NOX2 can be found in a primed state, a ready-to-go state which enhances its activation and thus ROS production (8, 12, 13). Neutrophil ROS production is primed by various mediators such as TNFα, GM-CSF, IL-8, and TLR agonists such as Lipopolysaccharides (LPS) and CL097 (8, 12–20). Physiological priming of the neutrophil NOX2 is believed to have many beneficial effects, such as efficient anti-bacterial and anti-fungal elimination (8, 21, 22). However, excessive priming of NOX2 results in excessive ROS production contributing to tissue damage involved in inflammatory diseases (8, 23–28). LPS is a main component of the outer membrane of gram-negative bacteria and it is released during bacterial infection. LPS is one of the most pathogenic molecules inducing immune cell activation and inflammation via TLR4 receptor (21, 29). LPS is known to induce NADPH oxidase priming in neutrophils by inducing NOX2 translocation to the membranes and p47^*phox*^ phosphorylation (14–17).

The peptidyl-prolyl *cis-trans* isomerase (PPIase), Pin1 is an enzyme which catalyzes the isomerization of prolyl peptide bonds from *cis-*conformation to *trans-*orientation (30). Pin1 recognizes a phosphor-Ser/Thr-Pro sequence and has been demonstrated to be a crucial regulator of many proteins phosphorylated on serine/threonine (31). Pin1 plays significant roles in a range of pathologies, including cancer, cardiovascular disease, and Alzheimer disease (32). We have shown that Pin1 was involved in TNF- and CL097 (a TLR7/8 agonist)-induced priming of NADPH oxidase in human neutrophils (18, 19, 33). However, the role of Pin1 in LPS-induced priming of NOX2 in neutrophils is not known. In this study we show that Pin1 is a key enzyme in LPS-induced priming of NOX2 in human PMN. Targeting Pin1 could be a new approach to treat inflammation and sepsis.

## Materials and Methods

### Reagents

Lipopolysaccaride (LPS) from E. Coli O111:B4 strain, Juglone, PiB, Phosphate Buffered Saline (PBS), Hanks' Balanced Salt Solution (HBSS), protease and phosphatase inhibitors were obtained from Sigma Aldrich (Saint Quentin Fallavier, France). Dextran T500 and Ficoll was from GE healthcare (Orsay, France). Sodium dodecyl-sulfate polyacrylamide (SDS-PAGE) and western blotting reagents were supplied by Bio-Rad (Hercules, CA, USA). The rabbit polyclonal antibodies against phospho-p47^*phox*^ sites (phospho-Ser345, phospho-Ser320, phospho-Ser304, phospho-Ser315, phospho-Ser328), p67^*phox*^, and p47^*phox*^ were produced by our lab as described elsewhere (18, 33). Anti-phospho(P)-ERK1/2, ERK1/2, P-p38, and p38 were from cell signaling Technology (Boston, MA, USA). HRP-conjugated goat anti-mouse were from Santa Cruz Biotechnology Inc. (Heidelberg, Germany).

### Ethics Statement

Neutrophils were isolated from healthy volunteers' venous blood with their signed informed consent. The collection and analyses of data were performed anonymously. All experiments were supported by the Inserm Institutional Review Board and ethics committee.

### Isolation of Human PMN

Neutrophils were isolated from blood of healthy volunteers as described previously (18, 33, 34). After hypotonic lysis of erythrocytes, the neutrophil pellets were collected and washed in PBS before cell counting. Viability was tested using Trypan Blue dye exclusion. This isolation method consistently yielded PMN with 96% pure and 99% viable.

### Luminol-Enhanced Chemiluminescence

To determine ROS production we used luminol-enhanced chemiluminescence method (33, 34). Neutrophils (2.5 × 10^5^) were resuspended in 0.5 mL of HBSS containing 10 μM of luminol with or without different concentrations of agents (PiB or juglone) for 20 min at 37°C, LPS was added for another 20 min; then fMLP (10^−7^ M) was used to stimulate the cells. Chemiluminescence was recorded using a luminometer (LB937; Berthold-Biolumat).

### Determination of CD11b-Expression and CD62L Shedding at the Neutrophil Surface

Neutrophils (10 × 10^6^ /ml) were incubated at 37°C in HBSS alone (control) or in the presence of PiB (50 μM) or Juglone (400 nM) for 30 min at 37°C. Samples were then incubated with LPS (1 μg/ml) or PBS (control) for another 20 min. A total of 100 μL of each sample was then stained with 10 μL of PE-conjugated anti-human CD11b monoclonal antibody (BD Biosciences, San Jose, CA) or 10 μL of fluorescein isothiocyanate (FITC)-conjugated anti-human CD62L monoclonal antibody for 30 min at room temperature in the dark. Cells were resuspended in 1% paraformaldehyde-PBS and kept on ice until flow cytometry. Non-specific antibody binding was determined on cells incubated with the same concentration of an irrelevant antibody of the same isotype. Forward and side scatter were used to identify the neutrophil population and to gate out other cells and debris in a FACS CantoII (BD Biosciences). The purity of the gated cells was assessed by using monoclonal anti-CD15 antibodies (BD Biosciences). The mean fluorescent intensity of ethidium, CD11b-positive cells and CD62L-positive cells was then determined in the neutrophil populations. Five thousand events per sample were analyzed, and all results were obtained with a constant photomultiplier gain value. Results were expressed as mean fluorescence intensity (MFI).

### Pin 1 Activity Assay

Pin1 activity was determined as previously described (18, 30, 33) with some modifications. In short, neutrophils were resuspended in an ice cold lysis buffer containing 50 mM HEPES pH 7.5, 0.25% CHAPS, 100 mM NaCl, 1 mM β-glycerophosphate, 5 mM NaF and 1 mM EGTA, at 10^7^/ml, and lysed with several a 2 ml-syringe pressures. The assay mixture contains 369 μL HEPES buffer (50 mM HEPES (pH 7.8), 25 μL (60 mg/ml) chymotrypsin solution (Sigma-Aldrich), 6 μL (6 mM) of the peptide Suc-Ala-Glu-Pro-phe-pNA (BACHEM), and 50 μL cell lysate (10^6^ cell equivalent). The absorbance change due to pNA release was monitored at 410 nm at 4°C by spectrophotometer (18, 30, 33) and the results were expressed as OD/min/1 million cells.

### SDS-PAGE and Western Blotting

Neutrophils (10^7^ cells in 500 μl of HBSS) were incubated with or without increasing LPS concentrations for 20 min at 37°C. The reaction was stopped by adding 125 μl of concentrated modified Laemmli sample buffer (5X) (35) containing 50 μg/mL pepstatin, 50 μg/mL leupeptin, 25 mM NaF, 12.5 mM Na3VO4, 12.5 mM EDTA, 12.5 mM EGTA, 6.25 mM p-NPP, and 50 μg/mL aprotinin. Samples were denatured in boiling water (100°C) for 3 min and stored at −80°C until use. Samples were thawed and sonicated for 10 s before use and then they were subjected to 10% SDS–PAGE with classical techniques (35). The separated proteins were transferred to nitrocellulose membranes (35). Nitrocellulose membranes were blocked with 5% non-fat dry milk in a mixture of tris-bufferd saline and tween-20. The membranes were incubated overnight at 4°C in solution containing specific relevant primary antibodies; anti-phospho-S345 (1:10,000), anti-phospho-S328 (1:2,500), anti-phospho-S304 (1:2,500), anti-phospho-S315 (1:2,500), anti-phospho-S320 (1:2,500), anti-phospho-ERK1/2 (1/2,000), anti-phospho-p38 (1:2,000), and p47^*phox*^(1:5,000) following by incubation in secondary antibodies (Santa Cruz, Heidelberg, Germany). Blots were visualized by using ECL Western blotting reagents (Amersham Pharmacia).

### Statistical Analysis

Experiments were repeated at least three times. All results are reported as means ± SEM. Significant differences (*p* < 0.05) were evaluated with Student's *t* tests and one-way ANOVA followed by Tukey's *post-hoc* test using Prism 8.0 software (GraphPad).

## Results

### LPS-Induced Priming of fMLP-Induced ROS Production in Human Neutrophils Is Impaired by Two Different Pin1 Inhibitors PiB and Juglone

**Figure 1:**
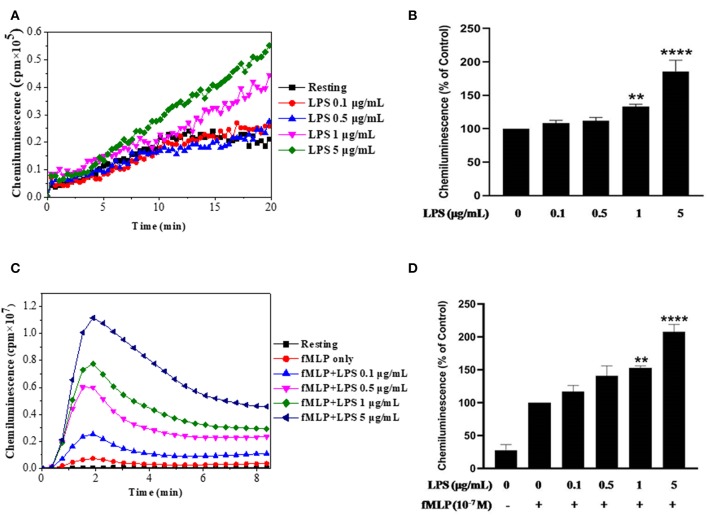
Effect of LPS alone or in combination with fMLP on ROS production by human neutrophils. **(A)** Human neutrophils (1 × 10^6^/ml) were incubated in HBSS without or with different concentrations of LPS for 20 min at 37°C. ROS production was measured using a luminol-amplified chemiluminescence technique. **(B)** Total chemiluminescence in each experimental condition is expressed as mean plus or minus SEM of 3 different experiments as compared to untreated cells (control 100%). **(C)** Human neutrophils (1 × 10^6^/ml) were incubated in the presence or absence of increasing concentrations of LPS for 20 min at 37°C, then stimulated with fMLP (10^−7^M). **(D)** Data are mean plus or minus SEM of 5 experiments as compared to fMLP only (control 100%) (^**^p < 0.05, ^****^p < 0.01).

In this study we used LPS from E. Coli O111:B4 strain, we wanted first to check its effect on ROS production by using the luminol-enhanced chemiluminescence assay, a very sensitive technique. Results show that LPS alone had no effect on ROS production by neutrophils at low concentrations and we found a weak but significant increase in ROS production starting from 1 μg/ml of LPS (Figures 1A,B). However, when neutrophils were treated by LPS and stimulated with fMLP (10^−7^ M), ROS production was markedly enhanced compared to neutrophils stimulated with fMLP alone (Figures 1C,D). The priming effect of LPS on fMLP-stimulated neutrophils is dose-dependent, and starts to be significant at 1 μg/ml in our conditions.

**Figure 2:**
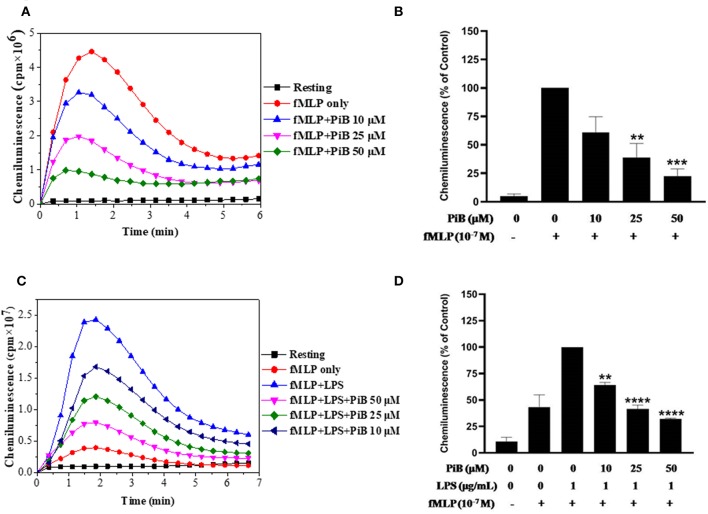
Effect of PiB, a Pin1 inhibitor on fMLP-induced and LPS-primed ROS production by human neutrophils. **(A)** Human neutrophils (1 × 10^6^/ml) were incubated in the absence or presence of increasing concentrations of PiB for 20 min at 37°C, then stimulated with fMLP (10^−7^ M). ROS production was measured with a luminol-amplified chemiluminescence technique. **(B)** Total chemiluminescence in each experimental condition is expressed as mean plus or minus SEM of 3 experiments. ^**^p < 0.05 and ^***^p < 0.01 as compared with fMLP alone (control 100%). **(C)** Human neutrophils (1 × 10^6^/ml) were incubated in the absence or presence of increasing concentrations of PiB for 20 min at 37°C, then LPS (1 μg/mL) was added for another 20 min before stimulation with fMLP (10^−7^ M). ROS production was measured with a luminol-amplified chemiluminescence technique. **(D)** Total chemiluminescence in each experimental condition is expressed as mean plus or minus SEM of 3 experiments. ^**^p < 0.05, ^****^p < 0.001 as compared with control (100%).

**Figure 3:**
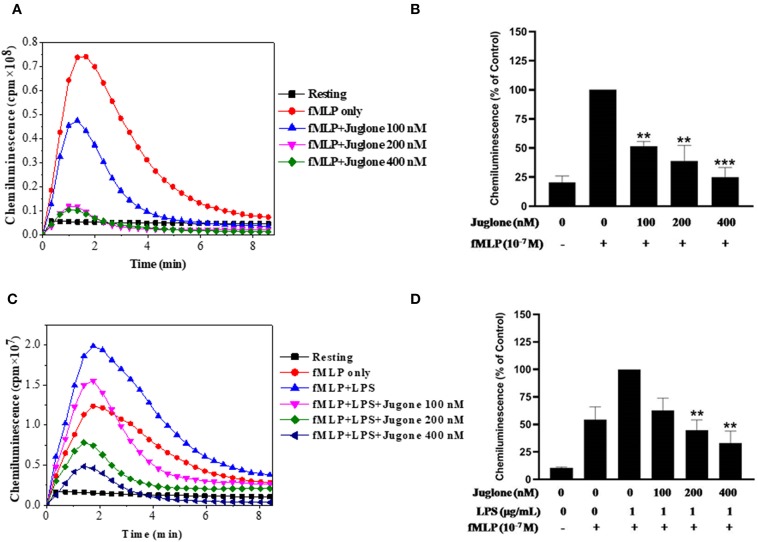
Effect of Juglone, a Pin1 inhibitor on fMLP-induced and LPS-primed ROS production by human neutrophils. **(A)** Human neutrophils (1 × 10^6^/ml) were incubated in the absence or presence of increasing concentrations of Juglone for 20 min at 37°C, then stimulated with fMLP (10^−7^ M). ROS production was measured with a luminol-amplified chemiluminescence technique. **(B)** Total chemiluminescence in each experimental condition is expressed as mean plus or minus SEM of 3 experiments. ^**^p < 0.05 and ^***^p < 0.001 as compared with fMLP alone (control 100%). **(C)** Human neutrophils (1 × 10^6^/ml) were incubated in the absence or presence of increasing concentrations of Juglone for 20 min at 37°C, then LPS (1 μg/mL) was added for another 20 min before stimulation with fMLP (10^−7^ M). ROS production was measured with a luminol-amplified chemiluminescence technique. **(D)** Total chemiluminescence in each experimental condition is expressed as mean plus or minus SEM of 3 experiments. ^**^p < 0.01 as compared with control (100%).

After confirming the priming effect of LPS on fMLP-induced ROS production in our experimental conditions, we next investigated the role of Pin 1 in LPS-induced priming of ROS production. In order to do so, we used two known Pin 1 selective inhibitors: PiB (36) and Juglone (37). Neutrophils were incubated with PiB (10–50 μM) or Juglone (100–400 nM) for 20 min, treated with LPS (0.1–5 μg/ml) for another 20 min, then stimulated with fMLP (10^−7^μM). ROS production was measured with luminol-enhanced chemiluminescence assay. Results show that PiB exhibited a significant inhibition of fMLP-induced ROS production in unprimed neutrophils (Figures 2A,B). In LPS primed cells, the inhibition was obtained at lower PiB concentrations (Figures 2C,D). Indeed at 25 μM, PiB was able to completely abolish the priming effect of LPS but did not completely inhibit fMLP-stimulated neutrophils. Juglone at 100–400 nM, showed a similar inhibition pattern as PiB on fMLP- and LPS-primed neutrophils (Figure 3). These results suggest a potential role of Pin 1 in fMLP-induced and LPS- primed ROS production in human neutrophils.

### Pin1 Inhibitors PiB and Juglone Do Not Inhibit LPS-Induced CD11b Plasma Membrane Translocation and CD62L Shedding

**Figure 4:**
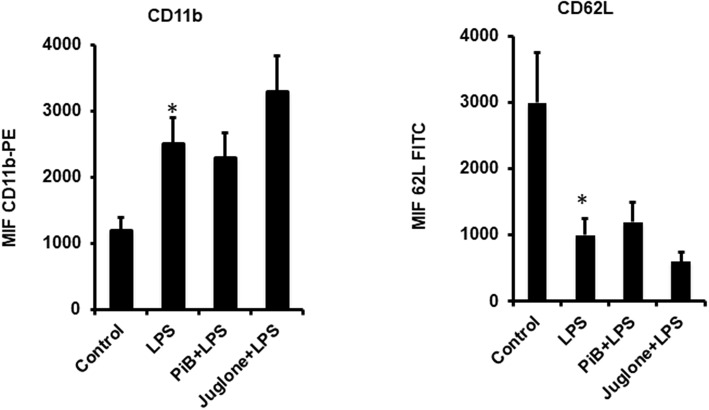
Effect of PiB and Juglone on LPS-induced CD11b expression on neutrophil plasma membrane and CD62L shedding. Human neutrophils (10 × 10^6^/ml) were incubated in the absence or presence of PiB (50 μM) or Juglone (400 nM) for 20 min at 37°C, then treated by LPS (1 μg/ml) for another 20 min. CD11b **(Left)** and CD62L **(Right)** expression at the plasma membrane was determined by flow cytometry using specific antibodies. Data are mean ± SEM of three experiments. ^*^p < 0.005 when LPS treated cells were compared to control untreated cells.

LPS is known to induce neutrophil degranulation, an other key neutrophil function (15, 16). We thus wanted to investigate the effect of Pin1 inhibitors on this function using flow cytometry to detect CD11b (a marker of specific and gelatinase marker) expression at the plasma membrane and CD62L plasma membrane shedding. Results show that LPS induced a clear CD11b expression at the plasma membrane and a clear shedding of CD62L compared to control untreated neutrophils (Figure 4). PiB did not affect CD11b expression nether CD62L shedding (Figure 4). However, Juglone did not inhibit these processes, rather it has an unexpected stimulatory effect. These results suggest that Pin1 is not involved in LPS-induced neutrophil degranulation.

### LPS Is Able to Induce Pin1 Activation in Human Neutrophils

**Figure 5:**
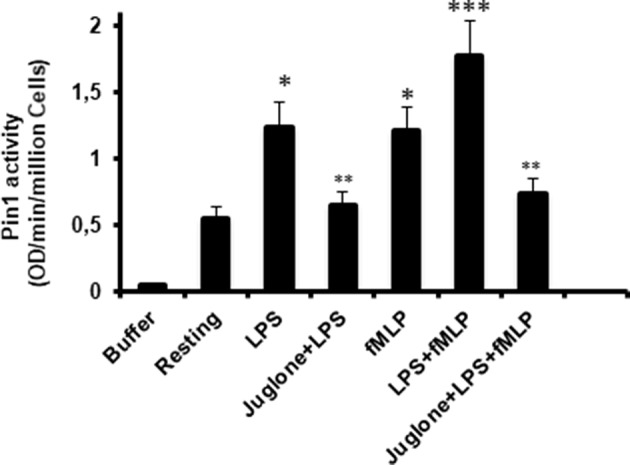
Pin1 is activated by LPS, fMLP, and LPS + fMLP in neutrophils and Juglone markedly decreased Pin 1 activity. Neutrophils were incubated in the absence and presence of Juglone (400 nM for 20 min) and then treated with LPS (5 μg/mL), fMLP (10^−7^ M), or LPS + fMLP before lysis. Pin1 activity was determined by measuring the absorbance of free pNA cleaved from Trp-Phe-Tyr-Ser (PO_3_H_2_)-Pro-Arg-pNA Suc at 410 nm. Results are expressed as Optical Density (OD)/min/million cells. Data are mean plus or minus SEM of 8 experiments. *p* < 0.01 as LPS and fMLP compared to resting cells (^*^); Cells treated with juglone compared to untreated cells (^**^); and LPS+fMLP as compared to LPS alone or fMLP alone (^***^).

To further investigate the implication of Pin1 in the priming effect of LPS on neutrophil ROS production, we investigated the effect of LPS on Pin1 activation. Neutrophils were incubated with LPS for 20 min, in the absence and presence of Juglone, then lysed. TNFα and fMLP were used as positive controls (18, 33). The activity of Pin 1 was determined by measuring the absorbance of free pNA resulted from the cleavage of Suc-Ala-Glu-Pro-Phe-pNA after it *cis to trans* conformational changes. Results presented in Figure 5 show that LPS strongly increased Pin1 activity (*P* < 0.0001 compared to resting cells). The activation effect of LPS on Pin1 was very similar to the one exhibited by fMLP (Figure 5) and TNFα (data not shown) which are strong enhancers of Pin 1 activity (33). Treatment of cells with Juglone markedly reduced LPS-induced activation of Pin 1, showing that this assay is specific for Pin1 (*P* < 0.001 compared to cells treated with LPS alone). Interestingly, Pin1 activity in neutrophils treated with both LPS + fMLP is higher than the activity in neutrophils treated with LPS alone or fMLP alone and Juglone inhibited this process. The results obtained suggest a key role of Pin 1 in LPS-induced priming of ROS production by human neutrophils in response to fMLP.

### LPS Induces Phosphorylation of p47*^*phox*^* Mainly on Serine 345, a Pin1 Binding Site

**Figure 6:**
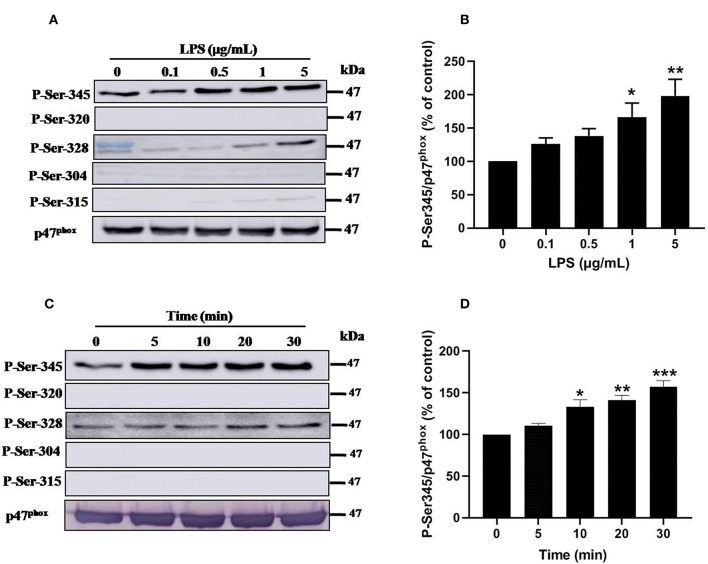
Effect of LPS on p47^*phox*^ phosphorylation in neutrophils. **(A)** Neutrophils (10 × 10^6^ cells/mL) were incubated with various concentrations of LPS (0, 0.1, 0.5, 1, 5 μg/mL) for 20 min at 37°C and the cells were lyzed. Proteins were analyzed by SDS-PAGE and western blot using anti-phospho-Ser-345, anti-phospho-Ser-320, anti-phospho-Ser-328, anti-phospho-Ser-304, anti-phospho-Ser-315, and anti-p47^*phox*^ antibodies. **(B)** Western blots from different experiments were scanned and quantified. The intensity of bands was expressed relative to total p47^*phox*^ amount. The cumulated data of phospho-Ser-345 is shown in the histogram as percentage to control (Resting 100%). **(C)** Neutrophils (10 × 10^6^ cells/mL) were incubated with LPS (1 μg/mL) for indicated times (0, 5, 10, 20, 30 min). Cells were analyzed by SDS-PAGE and immunoblotting with anti-phospho-Ser-345, anti-phospho-Ser-320, anti-phospho-Ser-328, anti-phospho-Ser-304, anti-phospho-Ser-315, and anti-p47^*phox*^ antibodies. **(D)** Western blots from different experiments for the kinetic effect were scanned and quantified. The intensity of bands was expressed relative to total p47^*phox*^ amount. Data are mean ± SEM of three or more separate experiments. ^*^p < 0.05, ^**^p < 0.01 and ^***^p < 0.001 as compared to control (100%).

Priming of the NADPH oxidase in neutrophils is controlled by different pathways, mainly the phosphorylation of p47^*phox*^ and the translocation of NOX2 from granules to the plasma membrane (8, 33). To further understand the mechanisms of LPS induced priming of ROS production by neutrophil, we studied the phosphorylation of different sites of p47^*phox*^. Results show that stimulation of neutrophils with LPS (0.1, 0.5, 1 and 5 μg/mL) induced a significant (*p* < 0.05) dose-dependent phosphorylation of p47^*phox*^ mainly on Ser345 and at lower level on Ser328 (Figures 6A,B). However, no phosphorylation effect was exerted on Ser304, Ser315, and Ser320 of p47^*phox*^. Kinetic study of LPS induced phophosrylation of p47^*phox*^ showed that LPS (1 μg/mL) rapidly induced phosphorylation Ser345 up to 30 min (Figures 6C,D). The phosphorylation of Ser328 was also weakly induced but not the phosphorylation of Ser304, 315, and 320 even at 30 min of incubation. These results clearly show that LPS induced p47^*phox*^ phosphorylation on Ser345, a binding site for Pin1 in human neutrophils (33, 38).

### LPS Induces Activation of p38MAPK and ERK1/2 Signaling Pathways in Human Neutrophils

**Figure 7:**
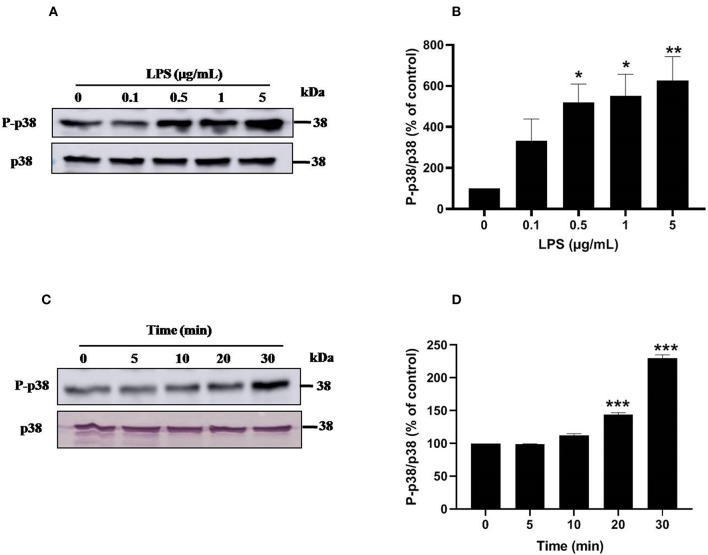
LPS induces p38 MAPK activation in human neutrophils. **(A)** Neutrophils (10 × 10^6^ cells/mL) were incubated with various concentration of LPS (0, 0.1, 0.5, 1, 5 μg/mL) for 20 min at 37°C. Cells were analyzed by SDS-PAGE and western blot using anti-phospho-p38 and anti-p38 antibodies. **(B)** Western blots from different experiments were scanned and quantified, total p38 were quantified by densitometry, and the intensity of phosphorylated p38 was corrected for the protein amount of p38. **(C)** Neutrophils (10 × 10^6^ cells/mL) were incubated with LPS (1 μg/mL) for indicated times (0, 5, 10, 20, 30 min). Cells were analyzed by SDS-PAGE and immunoblotting with anti-phospho-p38 and anti-p38 antibodies. **(D)** Western blots from different experiments for the kinetic effect were scanned and quantified, total p38 were quantified by densitometry, and the intensity of phosphorylated p38 was corrected for the protein amount of p38. Data are mean ± SEM of three or more separate experiments. *p* < 0.005 as compared to control (100%). ^*^p < 0.05, ^**^p < 0.01 and ^***^p < 0.001.

**Figure 8:**
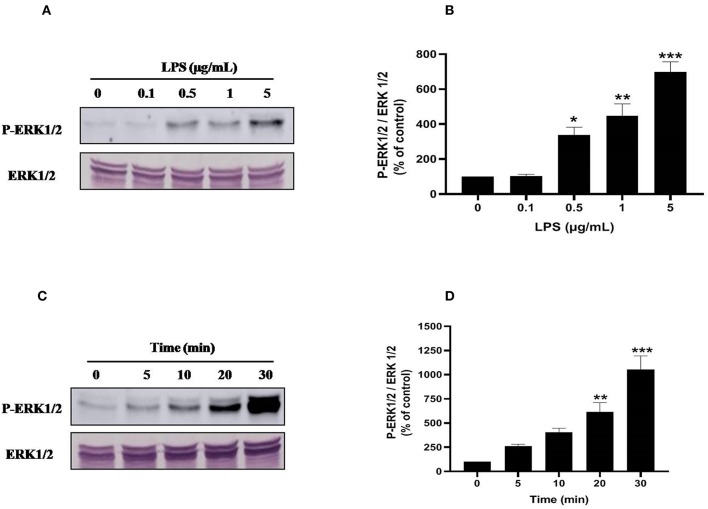
LPS induces ERK1/2 (p44/42 MAPK) activation in human neutrophils. **(A)** Neutrophils (10 × 10^6^ cells/mL) were incubated with various concentration of LPS (0, 0.1, 0.5, 1, 5 μg/mL) for 20 min at 37°C. Cells were analyzed by SDS-PAGE and western blot using anti-phospho-ERK1/2 and anti-ERK1/2 antibodies. **(B)** Western blots from different experiments were scanned, total ERK1/2 were quantified by densitometry, and the intensity of phosphorylated ERK 1/2 was corrected for the protein amount of ERK1/2. **(C)** Neutrophils (10 × 10^6^ cells/mL) were incubated with LPS (1 μg/mL) for indicated times (0, 5, 10, 20, 30 min). Cells were analyzed by SDS-PAGE and immunoblotting with anti-phospho-ERK1/2 and anti-ERK1/2 antibodies. **(D)** Western blots from different experiments for the kinetic effect were scanned and phosphorylated, total ERK1/2 were quantified by densitometry, and the intensity of phosphorylated ERK1/2 was corrected for the protein amount of ERK1/2. Data are mean ± SEM of three or more separate experiments. ^*^p < 0.05, ^**^p < 0.01 and ^***^p < 0.001 as compared to control (100%).

Ser345 of p47^*phox*^ is located in a MAPKinase phosphorylated site (-PGPQS(345)PG-) and is phosphorylated *in vitro* by p38MAPK and ERK1/2 (38). To investigate the kinase(s) involved in LPS-induced phosphorylation of Ser345 we first studied the phosphorylation (which reflects the activation) of different MAPKinases in LPS stimulated neutrophils. Our results show that neutrophils treatment with different concentrations of LPS induced a significant phosphorylation of p38MAPK in a dose-dependent manner. This phosphorylation was 6–8 folds higher than the basal one (Figures 7A,B). The kinetic study shows a retarded effect of LPS on p38MAPK (later than 10 min), with a maximum effect at 30 min (Figures 7C,D). Results also showed that LPS induced ERK1/2 phosphorylation in neutrophils in a concentration-dependent manner (Figures 8A,B). Likely to p38MAPK, kinetic study showed a retarded effect of LPS on ERK1/2 activation, starting at 10 min and which is potentiated till 30 min of treatment (Figures 8C,D). We also studied the effect of LPS on JNK1/2 phosphorylation in human neutrophils, results showed that LPS did not induce JNK phosphorylation (data not shown). Taken together, these results confirmed data from the literature (39, 40) and suggest that p38MAPK and ERK1/2 signaling pathways might be important effectors in LPS mediated priming of NOX2 in human PMN.

## Discussion

LPS or endotoxin is released by gram negative bacteria at sites of infection. It induced several neutrophil functions such as priming of superoxide production in combination to other stimuli such as the bacterial peptide fMLP. This LPS-induced priming of superoxide production was known since several years but the pathways involved in this process are unknown. Here we show that the peptidylprolyl cis/trans isomerase is a key enzyme of the LPS-induced NADPH oxidase priming.

This study, confirmed that LPS alone was not able to induce superoxide production as measured by luminol-amplified chemiluminescence as shown in Figure 1. At higher concentrations of LPS (>5 μg/ml) a weak superoxide production was observed but was not detected by an other specific technique such as cytochrome C reduction assay (data not shown). However, LPS even at low concentrations was able to enhance fMLP-induced superoxide production confirming its ability to prime this function.

To study the role of Pin1 in LPS-induced priming of superoxide production by neutrophils, we used two Pin1 inhibitors, PiB and Juglone as neutrophils are resistant to transfection. Both molecules inhibited LPS-induced priming of superoxide production and higher concentrations they inhibited also fMLP-induced activation. To check if Pin1 is also involved in LPS-induced degranulation and shedding to other neutrophil function, we tested the effect of PiB and Juglone on LPS-induced CD11b externalization from specific and gelatinase granules at the plasma membrane and the release of CD62L from the plasma membrane. PiB at high concentration (50 μM) did not have any effect, however Juglone stimulated this function. The reasons for these contrasting results are unknown and should be more investigated in the future. Juglone was shown to have some other effects and PiB is believed to be more selective for Pin1 (36, 41). CD11b is localized at the membrane of the same granules as gp91^*phox*^/NOX2 and p22^*phox*^ (15, 42–45), thus these results suggest also that PiB does not inhibit NOX2 translocation at the plasma membranes and Pin1 is not involved in this process.

To further investigate the role of Pin1 in LPS-treated neutrophils, we showed that LPS is able to increase Pin1 activation. Pin1 is constitutively active in resting cells in agreement with other reports and LPS was able to increase this activation in a comparable manner of TNF and fMLP (33). The pathways involved in the stimulation of Pin1 activity by TLR4 activation are unknown. It was shown that Pin1 is phosphorylated *in vitro* and in cells by protein kinase A (PKA) (46) and death-associated protein kinase 1 (DAPKinase 1) (47) and these phosphorylations inhibited its activity. To check if LPS is able to induce Pin1 dephosphorylation in human neutrophils, we tested different anti-phospho-Pin1 antibodies from different sources but the results were not conclusive. New anti-phospho-Pin1 antibodies are needed to study this pathway.

Upon activation Pin1 binds to phosphorylated Ser or Thr near a Pro. The NADPH oxidase component p47^*phox*^ is phosphorylated on Ser345 which is a Pin1 recognition motif (33, 38). We thus investigated the effect of LPS on p47^*phox*^ phosphorylation. Interestingly, LPS induced mainly the phosphorylation of Ser345 and at lesser extent the phosphorylation of Ser328. It is noteworthy that LPS alone did not induce the phosphorylation of Ser304, Ser315, and Ser320 required for NADPH oxidase activation explaining the lack of superoxide production with LPS alone. LPS also induced the phosphorylation of p38MAPKinase and ERK1/2 that are able to phosphorylate p47^*phox*^ on Ser345. We tested the effect of p38MAPK and ERK1/2 inhibitors which both inhibited p47phox phosphorylation on Ser345 (data not shown), suggesting that these MAPKinases converge to phosphorylate this site. In our previous work (33), we showed that Pin1 facilitates p47^*phox*^ phosphorylation on Ser328 and other serines upon fMLP stimulation. To check if in LPS-primed neutrophils, Pin1 is able to facilitate the phosphorylation of Ser328 we tested Juglone and PiB on this process. Results showed that Juglone and PiB were able to inhibit phosphorylation of p47^*phox*^ on Ser328 (data not shown), confirming our previous data.

**Figure 9:**
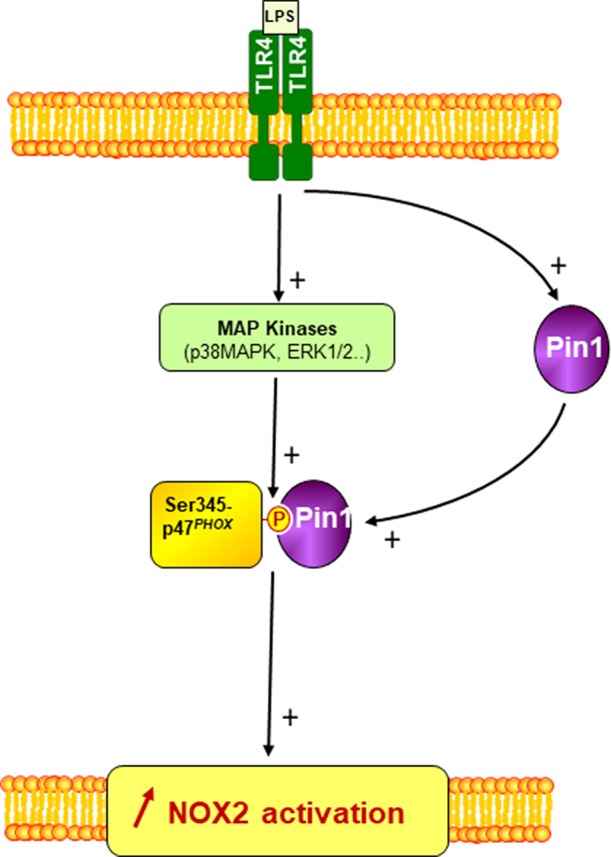
A scheme of the involvement of Pin1 and p47^*phox*^ phosphorylation in LPS-induced priming of NOX2. LPS via TLR4 induces Pin1 and MAP Kinases (p38MAPK and ERK1/2) activation in neutrophil cytosol. Active MAP Kinases phosphorylate p47^*phox*^ on serine 345 which is a binding site for activated Pin1. Pin1 binds to phosphorylated Ser345 and induces conformational changes of p47^*phox*^ to facilitate its phosphorylation by other Ser/Thr kinases and thus enhances NOX2 activation by a secondary agonist such as bacteria or fMLP.

LPS is the natural ligand of TLR4 (29). LPS induces neutrophil NADPH oxidase hyper-activation and activation of other immune cell functions mediating infection-induced inflammation and sepsis (27, 48). The results presented in this manuscript clearly show that Pin1 inhibitors inhibited LPS-induced priming of neutrophil NADPH oxidase activation, LPS induced Pin1 activation in human neutrophils and induced the phosphorylation of p47^*phox*^ on specific sites (Ser345 and 328) along with the activation of two MAPKinases p38 and ERK1/2. These events were presented in a scheme in Figure 9. Inhibitors of Pin1 at low concentrations could inhibit LPS-induced excessive ROS production at inflammatory site and might have anti-inflammatory effects while preserving the physiological ability of the bacterial N-formyl peptides to activate neutrophils.

## Data Availability Statement

The datasets generated for this study are available on request to the corresponding author.

## Author Contributions

ML, SB, MH-N, and CP designed and performed the experiments. PD, SY, and JE-B designed the experiments and analyzed the data. All authors contributed to writing the manuscript.

### Conflict of Interest

ML was employed by the company Yitong Food Industry Co. Ltd, China. The remaining authors declare that the research was conducted in the absence of any commercial or financial relationships that could be construed as a potential conflict of interest.

## References

[1] Nauseef WM Borregaard N. Neutrophils at work. Nat Immunol. (2014) 15:602–11. 10.1038/ni.2921 24940954

[2] Malech HL Deleo FR Quinn MT. The role of neutrophils in the immune system: an overview. Methods Mol Biol. (2014) 1124:3–10. 10.1007/978-1-62703-845-4_1 24504942 PMC6777345

[3] Mócsai A. Diverse novel functions of neutrophils in immunity, inflammation, and beyond. J Exp Med. (2013) 210:1283–99. 10.1084/jem.20122220 23825232 PMC3698517

[4] Witko-Sarsat V Rieu P Descamps-Latscha B Lesavre P Halbwachs-Mecarelli L. Neutrophils: molecules, functions and pathophysiological aspects. Lab Invest. (2000) 80:617–53. 10.1038/labinvest.3780067 10830774

[5] Borregaard N. Neutrophils, from marrow to microbes. Immunity. (2010) 33:657–70. 10.1016/j.immuni.2010.11.011 21094463

[6] Nauseef WM. How human neutrophils kill and degrade microbes: an integrated view. Immunol Rev. (2007) 219:88–102. 10.1111/j.1600-065X.2007.00550.x 17850484

[7] El-Benna J Dang PM Gougerot-Pocidalo MA Elbim C Phagocyte NADPH oxidase: a multicomponent enzyme essential for host defenses. Arch Immunol Ther Exp. (2005) 3:199–206. 15995580

[8] El-Benna J Dang PMC Hurtado-Nedelec M Marie JC Gougerot-Pocidalo MA. Priming of the neutrophil respiratory burst: role in host defense and inflammation. Imm Rev. (2016) 273:180–93. 10.1111/imr.12447 27558335

[9] Hampton MB Kettle AJ Winterbourn CC Inside the neutrophil phagosome: oxidants, myeloperoxidase, and bacterial killing. Blood. (1998) 12:3007–17. 10.1182/blood.V92.9.3007.421k47_3007_3017 9787133

[10] Belambri SA Rolas L Raad H Hurtado-Nedelec M Dang PM El-Benna J. NADPH oxidase activation in neutrophils: role of the phosphorylation of its subunits. Eur J Clin Invest. (2018) 48(Suppl. 2):e12951. 10.1111/eci.12951 29757466

[11] El-Benna J Dang PM Gougerot-Pocidalo MA Marie JC Braut-Boucher F. p47phox, the phagocyte NADPH oxidase/NOX2 organizer: structure, phosphorylation and implication in diseases. Exp Mol Med. (2009) 41:217–25. 10.3858/emm.2009.41.4.058 19372727 PMC2679237

[12] El-Benna J Dang PM Gougerot-Pocidalo MA. Priming of the neutrophil NADPH oxidase activation: role of p47phox phosphorylation and NOX2 mobilization to the plasma membrane. Semin Immunopathol. (2008) 30:279–89. 10.1007/s00281-008-0118-3 18536919

[13] Sheppard FR Kelher MR Moore EE McLaughlin NJ Banerjee A Silliman CC. Structural organization of the neutrophil NADPH oxidase: phosphorylation and translocation during priming and activation. J Leukoc Biol. (2005) 78:1025–42. 10.1189/jlb.0804442 16204621

[14] Forehand JR Pabst MJ Phillips WA Johnston RB Jr. Lipopolysaccharide priming of human neutrophils for an enhanced respirator burst. Role of intracellular free calcium. J Clin Invest. (1989) 83:74–83. 10.1172/JCI113887 2536046 PMC303645

[15] Leo FR Renee J McCormick S Nakamura N Apicella M Weiss JP Neutrophils exposed to bacterial lipopolysaccharide upregulate NADPH oxidase assembly. J Clin Invest. (1998) 101:455–63. 10.1172/JCI949 9435318 PMC508585

[16] Almkvist J Fäldt J Dahlgren C Leffler H Karlsson A. Lipopolysaccharide-induced gelatinase granule mobilization primes neutrophils for activation by galectin-3 and formylmethionyl-Leu-Phe. Infect Immun. (2001) 69:832–7. 10.1128/IAI.69.2.832-837.2001 11159975 PMC97959

[17] Hayashi F Means TK Luster AD. Toll-like receptors stimulate human neutrophil function. Blood. (2003) 102:2660–9. 10.1182/blood-2003-04-1078 12829592

[18] Makni-Maalej K Boussetta T Hurtado-Nedelec M Belambri SA Gougerot-Pocidalo MA El-Benna J. The TLR7/8 agonist CL097 primes N-formyl-methionyl-leucyl-phenylalanine-stimulated NADPH oxidase activation in human neutrophils: critical role of p47phox phosphorylation and the proline isomerase Pin1. J Immunol. (2012) 189:4657–65. 10.4049/jimmunol.1201007 23002436

[19] Makni-Maalej K Marzaioli V Boussetta T Belambri SA Gougerot-Pocidalo MA Hurtado-Nedelec M TLR8, but not TLR7, induces the priming of the NADPH oxidase activation in human neutrophils. J Leukoc Biol. (2015) 97:1081–7. 10.1189/jlb.2A1214-623R 25877926

[20] Hughes JE Stewart J Barclay GR Govan JR. Priming of neutrophil respiratory burst activity by lipopolysaccharide from *Burkholderia cepacia*. Infect Immun. (1997) 65:4281–7. 9317038 10.1128/iai.65.10.4281-4287.1997 PMC175614

[21] Picard C Puel A Bonnet M Ku CL Bustamante J Yang K. Pyogenic bacterial infections in humans with IRAK-4 deficiency. Science. (2003) 299:2076–9. 10.1126/science.1081902 12637671

[22] Karlsson A Markfjäll M Strömberg N Dahlgren C. *Escherichia coli*-induced activation of neutrophil NADPH-oxidase: lipopolysaccharide and formylated peptides act synergistically to induce release of reactive oxygen metabolites. Infect Immun. (1995) 63:4606–12. 7591113 10.1128/iai.63.12.4606-4612.1995 PMC173662

[23] Babior BM. Phagocytes and oxidative stress. Am J Med. (2000) 109:33–44. 10.1016/S0002-9343(00)00481-2 10936476

[24] Condliffe AM Kitchen E Chilvers ER. Neutrophil priming: pathophysiological consequences and underlying mechanisms. Clin Sci. (1998) 94:461–71. 10.1042/cs0940461 9682667

[25] Jacobi J Sela S Cohen HI Chezar J Kristal B. Priming of polymorphonuclear leukocytes: a culprit in the initiation of endothelial cell injury. Am J Physiol Heart Circ Physiol. (2006) 290:H2051–8. 10.1152/ajpheart.01040.2005 16387791

[26] Choi JC Jung JW Kwak HW Song JH Jeon EJ Shin JW. Granulocyte macrophage-colony stimulating factor (GM-CSF) augments acute lung injury via its neutrophil priming effects. J Korean Med Sci. (2008) 23:288–95. 10.3346/jkms.2008.23.2.288 18437014 PMC2526424

[27] Qian F Deng J Cheng N Welch EJ Zhang Y Malik AB. A non-redundant role for MKP5 in limiting ROS production and preventing LPS-induced vascular injury. EMBO J. (2009) 28:2896–907. 10.1038/emboj.2009.234 19696743 PMC2760111

[28] Nurcombe HL Bucknall RC Edwards SW. Neutrophils isolated from the synovial fluid of patients with rheumatoid arthritis: priming and activation *in vivo*. Ann Rheum Dis. (1991) 50:147–53. 10.1136/ard.50.3.147 1849716 PMC1004363

[29] Poltorak A He X Smirnova I Liu MY Van Huffel C Du X. Defective LPS signaling in C3H/HeJ and C57BL/10ScCr mice: mutations in Tlr4 gene. Science. (1998) 282:2085–8. 10.1126/science.282.5396.2085 9851930

[30] Yaffe MB Schutkowski M Shen M Zhou XZ Stukenberg PT Rahfeld JU. Sequence-specific and phosphorylation-dependent proline isomerization: a potential mitotic regulatory mechanism. Science. (1997) 278:1957–60. 10.1126/science.278.5345.1957 9395400

[31] Liou YC Zhou XZ Lu KP. Prolyl isomerase Pin1 as a molecular switch to determine the fate of phosphoproteins. Trends Biochem Sci. (2011) 36:501–14. 10.1016/j.tibs.2011.07.001 21852138 PMC3185210

[32] Lee TH Pastorino L Lu KP. Peptidyl-prolyl cis-trans isomerase Pin1 in ageing, cancer and Alzheimer disease. Expert Rev Mol Med. (2011) 13:e21. 10.1017/S1462399411001906 21682951

[33] Boussetta T Gougerot-Pocidalo MA Hayem G Ciappelloni S Raad H Arabi Derkawi R. The prolyl isomerase Pin1 acts as a novel molecular switch for TNF-alpha-induced priming of the NADPH oxidase in human neutrophils. Blood. (2010) 116:5795–802. 10.1182/blood-2010-03-273094 20956805 PMC3031377

[34] Hurtado-Nedelec M Makni-Maalej K Gougerot-Pocidalo MA Dang PM El-Benna J. Assessment of priming of the human neutrophil respiratory burst. Methods Mol Biol. (2014) 1124:405–12. 10.1007/978-1-62703-845-4_23 24504964

[35] Belambri SA Dang PM El-Benna J. Evaluation of p47phox phosphorylation in human neutrophils using phospho-specific antibodies. Methods Mol Biol. (2014) 1124:427–33. 10.1007/978-1-62703-845-4_25 24504966

[36] Uchida T Takamiya M Takahashi M Miyashita H Ikeda H Terada T. Pin1 and Par14 peptidyl prolyl isomerase inhibitors block cell proliferation. Chem Biol. (2003) 10:15–24. 10.1016/S1074-5521(02)00310-1 12573694

[37] Hennig L Christner C Kipping M Schelbert B Rücknagel KP Grabley S. Selective inactivation of parvulin-like peptidyl-prolyl cis/trans isomerases by juglone. Biochemistry. (1998) 37:5953–60. 10.1021/bi973162p 9558330

[38] Dang PM Stensballe A Boussetta T Raad H Dewas C Kroviarski Y. A specific p47phox -serine phosphorylated by convergent MAPKs mediates neutrophil NADPH oxidase priming at inflammatory sites. J Clin Invest. (2006) 116:2033–43. 10.1172/JCI27544 16778989 PMC1479423

[39] Nick JA Avdi NJ Gerwins P Johnson GL Worthen GS. Activation of a p38 mitogen-activated protein kinase in human neutrophils by lipopolysaccharide. J Immunol. (1996) 156:4867–75. 8648136

[40] Nolan B Duffy A Paquin L De M Collette H Graziano CM. Mitogen-activated protein kinases signal inhibition of apoptosis in lipopolysaccharide-stimulated neutrophils. Surgery. (1999) 126:406–12. 10.1016/S0039-6060(99)70185-6 10455914

[41] Ahmad T Suzuki YJ. Juglone in Oxidative Stress and Cell Signaling. Antioxidants. (2019) 8:E91. 10.3390/antiox8040091 30959841 PMC6523217

[42] Ward RA Nakamura M McLeish KR. Priming of the neutrophil respiratory burst involves p38 mitogen-activated protein kinase-dependent exocytosis of flavocytochrome b558-containing granules. J Biol Chem. (2000) 275:36713–9. 10.1074/jbc.M003017200 10976103

[43] Uriarte SM Rane MJ Luerman GC Barati MT Ward RA Nauseef WM. Granule exocytosis contributes to priming and activation of the human neutrophil respiratory burst. J Immunol. (2011) 187:391–400. 10.4049/jimmunol.1003112 21642540 PMC3582343

[44] Faurschou M Borregaard N. Neutrophil granules and secretory vesicles in inflammation. Microbes Infect. (2003) 5:1317–27. 10.1016/j.micinf.2003.09.008 14613775

[45] Bai J Tang L Lomas-Neira J Chen Y McLeish KR Uriarte SM. TAT-SNAP-23 treatment inhibits the priming of neutrophil functions contributing to shock and/or sepsis-induced extra-pulmonary acute lung injury. Innate Immun. (2015) 21:42–54. 10.1177/1753425913516524 24391146 PMC4092048

[46] Lu PJ Zhou XZ Liou YC Noel JP Lu KP. Critical role of WW domain phosphorylation in regulating phosphoserine binding activity and Pin1 function. J Biol Chem. (2002) 277:2381–4. 10.1074/jbc.C100228200 11723108

[47] Lee TH Chen CH Suizu F Huang P Schiene-Fischer C Daum S. Death-associated protein kinase 1 phosphorylates Pin1 and inhibits its prolyl isomerase activity and cellular function. Mol Cell. (2011) 42:147–59. 10.1016/j.molcel.2011.03.005 21497122 PMC3088080

[48] Pillay J Ramakers BP Kamp VM Loi AL Lam SW Hietbrink F. Functional heterogeneity and differential priming of circulating neutrophils in human experimental endotoxemia. J Leukoc Biol. (2010) 88:211–20. 10.1189/jlb.1209793 20400675

